# Cannabis use among Navy personnel in Sri Lanka: a cross sectional study

**DOI:** 10.1186/s13104-016-1988-4

**Published:** 2016-03-17

**Authors:** Varuni Asanka de Silva, Nicholas Jayasekera, Raveen Hanwella

**Affiliations:** Department of Psychological Medicine, Faculty of Medicine, University of Colombo, Kynsey Road, Colombo 08, Sri Lanka; Surgeon Rear Admiral, Director General Health Services, Sri Lanka Navy, Colombo, Sri Lanka; Department of Psychological Medicine, Faculty of Medicine, University of Colombo, Colombo 08, Sri Lanka

**Keywords:** Cannabis, Military, Substance use, Sri Lanka, Combat

## Abstract

**Background:**

Prevalence of cannabis use among military populations vary. There is evidence that drug use is associated with combat exposure and PTSD. The objective of the study was to assess the prevalence of cannabis use among Sri Lanka Navy (SLN) personnel and to identify any relationship with cannabis use and combat exposure.

**Methods:**

This cross sectional study was carried out among representative samples of SLN Special Forces (Special Boat Squadron) and regular forces deployed in combat areas. Both Special Forces and regular forces were selected using simple random sampling. Personnel who had served continuously in combat areas during the 1 year period prior to end of combat operations were included in the study. Cannabis use was defined as smoking cannabis at least once during the past 12 months.

**Results:**

The sample consisted of 259 Special Forces and 412 regular navy personnel. Prevalence of cannabis use was 5.22 % (95 % CI 3.53–6.9). There was no significant difference in prevalence of cannabis use among Special Forces personnel compared to regular forces. Cannabis use was significantly higher in the age group 18–24 years [OR 4.42 (95 % CI 2.18–8.97)], personnel who were never married [OR 2.02 (95 % CI 0.99–4.12)], or had an educational level less than GCE O’Level [OR 4.02 (95 % CI 1.17–13.78)]. There was significant association between cannabis use and hazardous alcohol use [adjusted OR 5.47 (95 % CI 2.65–11.28)], PTSD [adjusted OR 4.20 (95 % CI 1.08–16.38)], GHQ caseness [adjusted OR 2.83 (95 % CI 1.18–6.79)] and multiple somatic complaints [adjusted OR 3.61 (95 % CI 1.5–8.7)]. Cannabis use was not associated with smoking. Risk of cannabis use was less in those who had seen dead or wounded [adjusted OR 0.42 (95 % CI 0.20–0.85)]. Experiencing hostility from civilians was the only combat exposure that significantly increased the risk of cannabis use [adjusted OR 4.06 (95 % CI 1.06–15.56)].

**Conclusions:**

Among Sri Lanka Navy personnel exposed to combat cannabis use was significantly associated with hazardous alcohol use but not smoking. PTSD and other adverse mental health outcomes were associated with an increased risk of cannabis use. Exposure to combat was not associated with increased risk of cannabis use.

## Background

Cannabis also known as marijuana is an illicit psychoactive substance derived from the Cannabis sativa plant. Regular cannabis use is associated with cannabis dependence syndrome. Cannabis users are also more likely to use other illicit drugs. Studies in high income countries show that the pattern of drug initiation starts with alcohol and tobacco, followed by cannabis, and then other illicit drugs [1]. Cannabis use impairs cognitive and behavioural functions, especially for sustained-attention tasks [2, 3]. It also increases the risk of mental disorders particularly psychoses [4–6].

Cannabis use is increasing in both developed and developing countries [4]. The prevalence varies widely from 0.5–42 % depending on the population [7]. According to the National Epidemiologic Survey on alcohol and related conditions, in the United States, the lifetime prevalence of cannabis is 8.4 % in males and 4.3 % in females [8]. In Europe, among patients presenting to emergency rooms with acute drug toxicity, cannabis was the third commonest drug used after heroin and cocaine [9].

Several studies have shown an association between exposure to trauma and substance use. The National Comorbidity Survey found that one third of individuals with lifetime post traumatic stress disorder (PTSD) had lifetime substance use disorder [10]. This association is seen among both civilians and combat veterans [11]. Increased prevalence of PTSD has been demonstrated in several groups such as community dwelling adolescents with alcohol dependence, treatment seeking male substance users and non treatment seeking users [11]. These groups were exposed to physical or sexual assault or crime victimization [12].

The comorbidity rates for PTSD and substance use are highest for male combat veterans [11]. Among veterans with PTSD the rates of comorbid drug and alcohol use are much higher than in the general population. Prevalence of cannabis use among military populations vary. In the French army 18.5 % reported using cannabis at least once in the past 12 months and 8.1 % reported regular use (smoking at least 10 joints per month) [13]. Among Canadian Forces 14 % reported cannabis use [14]. Among military police in Brazil, lifetime use of cannabis was 8.1 % [15]. Khat is the commonest drug used by Somali combatants followed by cannabis (10.7 %) [16]. However low rates of drug use have been reported in some military personnel returning from combat duty. Among Persian gulf war veterans 2–3 % reported drug problems 6 years after return [17]. Low rates may be due to the fact that cannabis use is illegal in the military [18].

From 2006 to 2009 the Sri Lanka Defense Forces were engaged in combat operations. During this period 190 officers and 5700 other ranks of the Sri Lanka Army and 485 personnel from the SLN were killed and 27,000 injured [19]. Both Special forces and regular forces were exposed to traumatic events. More than 60 % in both groups had seen dead or injured persons. More than 80 % of the Special Forces reported discharge of weapons in direct combat compared to 26.7 % of the regular forces. Among the Special Forces, 81.5 % had engaged in combat with enemy vessels compared to 29.4 % of the regular forces [19].

Although there is evidence that drug use is associated with PTSD there is little evidence that it is associated with exposure to trauma per se. To investigate if use of cannabis in military personnel is associated with exposure to trauma we looked at cannabis use in the Sri Lanka Navy personnel deployed in combat areas.

## Methods

The study methods are described in detail in a previous publication [19]. The data was collected as part of a study comparing the mental health status of Special Forces personnel with regular forces of the Sri Lanka Navy (SLN). Data collection commenced 3 months after combat operations ended in 2009.

This cross sectional study was carried out among representative samples of SLN Special Forces and regular forces deployed in combat areas. Both Special Forces and regular forces were selected using simple random sampling. The sample of SLN Special Forces was selected from the Special Boat Squadron. The sample size was calculated to detect an odds ratio of 2.0 for disorders with an estimated prevalence of 15 %, a power of 90 % and confidence of 95 % (two tailed). The required sample size was 240 in each group. The sample size was increased by 15 % to adjust for nonresponse. The comparison group (regular forces) was oversampled to include more combat troops. The sampling frames used were the lists of personnel from the navy central data base. Samples were selected using computer generated random numbers. Participation was voluntary. The response rate was 93.8 %. The rate of missing values for individual items in the survey was about 10 %.

Only personnel who had served continuously in combat areas during the 1 year period prior to end of combat operations were included in the study. The sample included only males. A total of 259 Special Forces and 412 regular navy personnel were recruited to the study.

### Outcome measures

The 28 page questionnaire used in the study “Health of UK military personnel deployed to the 2003 Iraq war” was used as the data collection instrument [20]. Permission was obtained from the authors for the use of the questionnaire. Mental health outcomes were measured using several scales. Case definitions used were same as those in the study of UK personnel deployed to Iraq [20]. Symptoms of common mental disorder were identified using the General Health Questionnaire 12 (GHQ-12) and cases were defined as individuals scoring 4 or more. PTSD was diagnosed using the 17 item National Centre for PTSD checklist civilian version (PCL-C) and cases were defined as individuals scoring 50 or more. Fatigue was assessed using the 13 item Chalder fatigue scale and cases were defined as individuals scoring 4 or more [21]. Hazardous alcohol use was identified using the WHO alcohol use disorder identification test (AUDIT) with individuals scoring 8≥ identified as cases. Multiple physical symptoms were elicited using a checklist of symptoms and cases were defined as individuals with 10 or more symptoms. This case definition represents the top decile of this sample. Cannabis use was defined as smoking cannabis at least once within the past 12 months.

### Ethical approval

Ethical clearance was obtained from the Ethics Review Committee of the Faculty of Medicine, University of Colombo. Participation was voluntary and written informed consent was obtained from all participants. The questionnaire did not identify the participants by name.

### Statistical analysis

Prevalence of cannabis use was calculated according to demographic variables. Association between cannabis use and combat exposure was explored using multiple logistic regression analyses which adjusted for demographic variables and service type. Statistical analysis was carried out using SPSS version 13.0 for Windows.

## Results

### Study sample

The sample consisted of 259 Special Forces and 412 regular navy personnel [19]. The mean age of the sample was 27.6 years (SD 5.02). Of the sample 49.0 % were single, 49.6 % were married and 0.3 % were previously married. One third of the sample (35.2 %) were engaged in combat duty, 29.1 % served on board naval vessels and 35.3 % were engaged in non-combat duties which included medical, logistic, engineering, communication and administrative roles.

### Prevalence of cannabis use

Cannabis use according to demographic characteristics are shown in Table 1. Overall prevalence of cannabis use was 5.22 % (95 % CI 3.53–6.9). There was no significant difference in prevalence of cannabis use among Special Forces personnel compared to regular forces. [OR 0.94 (95 % CI 0.46–1.89)]. Personnel aged 18–24 years had a significantly higher risk of cannabis use compared to those aged ≥25 years [OR 4.42 (95 % CI 2.18–8.97)]. Personnel who were never married were more likely to be cannabis users [OR 2.02 (95 %CI 0.99–4.12)]. Those with an educational level less than GCE O’ Level were more likely to use cannabis than personnel with educational level of GCE A’ Level or higher [OR 4.02 (95 % CI 1.17–13.78)].

**Table 1:** Cannabis use according to demographic characteristic

	Cannabis use prevalence (95 % CI)	Unadjusted OR (95 % CI)
Service type^a^		
Special forces	5.02 (2.34–7.70)	0.94 (0.46–1.89)
Regular forces	5.34 (3.16–7.52)	1.0
Age (years)		
18–24	11.1 (6.7–15.53)	4.42 (2.18–8.97)
≥25	2.75 (1.27–4.23)	1.0
Marital status		
Never married	6.91 (4.17–9.64)	2.02 (0.99–4.12)
Married/divorced	3.55 (1.57–5.53)	1.0
Educational status		
<GCE O’level	8.2 (4.73–11.66)	4.02 (1.17–13.78)
GCE O level	4.15 (1.84–6.47)	1.95 (0.54–7.02)
GCE A level or higher	2.17 (0.29–4.64)	1.0

### Cannabis use and exposure to trauma

Association between ten items measuring combat exposure and cannabis use was assessed using logistic regression analysis (Table 2). Combat exposure such as direct combat, thoughts that the person might be killed, coming under small arms or mortar fire were not associated with cannabis use. Risk of cannabis use was less in those who had seen dead or wounded [adjusted OR 0.42 (95 % CI 0.20–0.85)]. Experiencing hostility from civilians was the only combat exposure that was significantly associated with increased cannabis use [adjusted OR 4.06 (95 % CI 1.06–15.56)]. Since there were significant differences in cannabis use according to age, marital status and educational status we adjusted for these variables and service type. After adjusting association between cannabis use and seeing dead or wounded, experienced hostility from civilians remained (Table 3) significant.

**Table 2:** Association between cannabis use and combat experience

Combat exposure	Cannabis users (N = 35)	Non users (N = 636)	Unadjusted OR	Adjusted OR^a^
Discharged weapon in direct combat	19 (54.3)	316 (49.7)	1.20 (0.61–2.38)	1.24 (0.54–2.85)
Thought might be killed	17 (48.6)	270 (42.5)	1.28 (0.65–2.53)	1.38 (0.69–2.78)
Seeing dead or wounded	16 (45.7)	449 (70.6)	0.35 (0.18–0.70)	0.42 (0.20–0.85)
Handled bodies	10 (28.6)	286 (45.0)	0.49 (0.23–1.04)	0.62 (0.28–1.35)
Aided wounded	9 (25.7)	240 (37.7)	0.57 (0.27–1.24)	0.76 (0.34–1.70)
Came under small arm fire	11 (31.4)	246 (38.7)	0.73 (0.35–1.51)	1.03 (0.46–2.30)
Came under mortar, missile, artillery fire	6 (17.1)	223 (35.1)	0.38 (0.16–0.94)	0.54 (0.21–1.38)
Experienced landmine strikes	0 (0)	25 (3.9)	–	–
Experienced hostility from civilians	3 (8.6)	15 (2.4)	3.88 (1.07–14.01)	4.06 (1.06–15.56)
Involved in combat with enemy vessels	14 (40.0)	318 (50.0)	0.67 (0.33–1.33)	0.70 (0.30–1.60)

^a^Adjusted for age, marital status, education and service type

**Table 3:** Association between cannabis use and mental health outcomes

Mental health outcome	Cannabis users (N = 35)	Non users (N = 636)	Unadjusted OR	Adjusted OR^a^
Common mental disorders	8 (22.9)	71 (11.2)	2.36 (1.03–5.39)	OR 2.83 (95 % CI 1.18–6.79)
PTSD	3 (8.6)	13 (2.0)	4.49 (95 % CI 1.22–16.56)	4.20 (1.08–16.38)
Fatigue	6 (17.1)	84 (13.2)	1.36 (95 % CI 0.55–3.37)	1.94 (95 % CI 0.74–5.08)
Multiple somatic symptoms	8 (22.9)	62 (9.7)	2.74 (1.20–6.30)	3.61 (95 % CI 1.50–8.7)
Hazardous alcohol use	16 (45.7)	97 (15.3)	4.73 (2.35–9.53)	OR 5.47 (95 % CI 2.65–11.28)
Smoking	9 (25.7)	111 (17.5)	1.63 (0.74–3.59)	OR 1.69 (95 % CI 0.75–3.79)

^a^Adjusted for age, marital status, education and service type

Of the different types of combat exposure, experiencing hostility from civilians [OR 9.8 (95 % CI 2.54–38.2)], feeling he might die [OR 3.02 (95 % CI 1.04–8.79)] and becoming injured from land mines [OR 6.64 (95 % CI 1.76–24.99)] were significantly associated with PTSD.

### Association with mental health outcomes

Cannabis use was significantly associated with PTSD [adjusted OR 4.20 (95 % CI 1.08–16.38)], GHQ caseness [adjusted OR 2.83 (95 % CI 1.18–6.79)] and multiple somatic complaints [adjusted OR 3.61 (95 % CI 1.5–8.7)]. These association remained even after adjusting for demographic variables. Fatigue was not significantly associated with cannabis use (Table 3).

### Cannabis and other substance use

There was significant association between cannabis use and hazardous alcohol use (total score of ≥8 in the AUDIT scale) [adjusted OR 5.47 (95 % CI 2.65–11.28)]. Current smoking was not significantly associated with cannabis use [adjusted OR 1.69 (95 % CI 0.75–3.79)].

## Discussion

This study provides information about cannabis use in a military population in Sri Lanka. It explores the association of cannabis use with combat exposure and mental health. The prevalence of cannabis use was 5.22 % (95 % CI 3.53–6.9) among SLN personnel. Younger age (18–24 years), personnel who were never married and those with an educational level less than GCE O’Level were more likely to use cannabis. Cannabis use was significantly associated with hazardous alcohol use but not smoking. Except for experiencing hostility from civilians, other types of combat exposure were not associated with an increased risk of cannabis use. Cannabis use was significantly associated with PTSD, GHQ caseness and experiencing multiple somatic complaints.

The sample consisted of Special Forces and regular forces personnel exposed to combat. We have previously reported that the prevalence of common mental disorders (11.8 %), PTSD (2.4 %), fatigue (13.4 %), multiple physical symptoms (10.4 %) and hazardous alcohol use in the SLN personnel was less than that in United Kingdom (UK) and United States (US) personnel deployed in Iraq and Afghanistan [19, 20, 22–24]. Despite higher exposure to potentially traumatic events Special Forces had less mental health problems compared to regular forces. We found that there was no significant difference in cannabis use between Special Forces and regular forces.

In military populations combat induced PTSD is associated with substance abuse [25]. This may be because chronic substance users are more vulnerable to developing PTSD or because people with PTSD use psychoactive substances as a means of self medication [11]. We too found that cannabis use was significantly associated with PTSD.

We found that experiencing hostility from civilians was the only combat exposure that was significantly associated with cannabis use. To understand the association between combat exposure, PTSD and cannabis use we looked at the relationship between these variables. Of the different types of combat exposure, experiencing hostility from civilians, feeling one might die and injury from land mines were significantly associated with PTSD. Therefore hostility from civilians may be a highly traumatic experience which increases the risk of PTSD and also cannabis use. However it must be noted that only a total of 18 individuals experienced hostility by civilians and of these only three were cannabis users. Injury following land mine blasts and feeling one might die are also highly traumatic experiences which increased the risk of PTSD, but these were not associate with cannabis use. There is evidence that PTSD rather than combat stress per se is associated with substance use [25]. Our findings support this.

Cannabis may be used to cope with symptoms of PTSD. Some states in the United States of America have approved the use of medical marijuana for PTSD. A study has reported that 23 % of patients seeking medical cannabis for the first time screened positive for PTSD [30]. Greater severity of PTSD was associated with more frequent cannabis use [32]. Those with PTSD were more likely to seek medical cannabis than those without PTSD. There is evidence that cannabis is used to cope with symptoms such as poor sleep and intrusive thoughts [31].

Seeing dead or wounded were associated with a significantly lower risk of cannabis use. We have previously reported that significantly more Special Forces personnel had reported seeing dead or wounded [19]. Lower rates of mental health problems among Special Forces may explain the lower associated risk of cannabis use.

According to the gateway theory, tobacco or alcohol use leads to cannabis use, and cannabis users more likely to go on to use heroin and cocaine [1]. However the evidence regarding the gateway theory is inconsistent and we too did not find a significant association between cannabis use and smoking [26]. In this sample the prevalence of smoking 17.9 % was lower than that reported among the general population of 29.9 % in urban areas and 24.4 % in rural areas [27, 28]. The anti smoking policy which was in force in the SLN at that time restricted access to cigarettes. However there was significant association between hazardous alcohol use and cannabis use. There is evidence that hazardous alcohol use is associated with PTSD [12].

The General Health Questionnaire is a scale used to identify psychological morbidity in non-psychiatric settings. Our study found that cannabis users were more likely to be identified as cases based on the GHQ score. This association disappeared when we adjusted for hazardous alcohol use suggesting that hazardous alcohol use acts as a confounding factor in the aetiology of psychological morbidity.

In the absence of data on cannabis use among the general population in Sri Lanka, it is not possible decide if the rate of cannabis use in this military population is different to that of the general population. Since this sample consisted only of males and a high proportion were young and unmarried, which are factors associated with illicit drug use, we can expect the overall prevalence in this group to be higher than in a general population sample. The prevalence in this sample was higher than among a cohort of mentally ill patients in Sri Lanka [29]. However that study may have underestimated use as it relied on patient records for identification of cannabis use.

We have previously reported that the prevalence of hazardous alcohol use and smoking in this sample are less than that reported in US and UK military personnel [19]. Prevalence of cannabis use too is less than that reported among French, Canadian, Brazilian and Somalian military personnel [13–16]. Prevalence of probable PTSD was 1.9 % in the Special Forces and 2.7 % in the regular forces [19]. Since there is evidence that substance use disorders are associated with PTSD, the low rate of PTSD may explain the low rate of cannabis and alcohol use in this sample. Access to cannabis also may have been restricted because the personnel were deployed in combat areas and because interactions with civilians and other means of acquiring cannabis were limited.

The main limitation in our study was that self reports were used to identify cannabis use. Under reporting is known to occur with self reports on substance use. Under reporting of cannabis use among our sample is a distinct possibility because cannabis is an illicit drug. We also did not assess the frequency and quantity of cannabis use. Despite this limitation, this study provides data on cannabis use in Sri Lanka and also supports previous findings that there is no significant association between cannabis use and combat exposure.

## Conclusions

The prevalence of cannabis use was lower among Sri Lanka Navy personnel than that reported among military personnel from other countries. Younger age, personnel who were never married and those with an educational level less than GCE O’Level were more likely to use cannabis. Cannabis use was significantly associated with hazardous alcohol use but not smoking. PTSD and other adverse mental health outcomes were associated with an increased risk of cannabis use. Exposure to combat per se was not associated with an increased risk of cannabis use.

## Abbreviations

SLN: Sri Lanka Navy
AUDIT: alcohol yse disorder identification test
GHQ: General Health Questionnaire
